# Supplementary material for: Aurora A depletion reveals centrosome-independent polarization mechanism in Caenorhabditis elegans
Source: eLife. 2019 Feb 26;8:e44552. doi: 10.7554/eLife.44552 (PMC6417861; doi:10.7554/eLife.44552)
Supplement: Supplementary file 1. [file elife-44552-supp1.docx]

| **List of wormstrains** | | |
| --- | --- | --- |
| **genotype of original strains** | **strain** | **Reference** |
| wild type, Bristol | N2 | [Brenner, 1974](#_ENREF_3) |
| *sas-7(or1940[gfp::sas-7])III* | EU2873 | [Sugioka et al., 2017](#_ENREF_15) |
| *nmy-2(ges6(nmy-2::RFP-T+unc-119(+)))I* | SWG008 | [Nishikawa, Naganathan, Julicher, & Grill, 2017](#_ENREF_9) |
| *par-2(it328[gfp::par-2])III* | KK1273 | Ken Kemphues, unpublished  https://cgc.umn.edu/strain/KK1273 |
| *par-6(it319[par-6::gfp])I* | KK1248 | Ken Kemphues, unpublished  https://cgc.umn.edu/strain/KK1248 |
| *par-2(it315[mcherry::par-2])III* | KK1254 | Ken Kemphues, unpublished  https://cgc.umn.edu/strain/KK1254 |
| *pkc-3(it309[gfp::pkc-3])II* | KK1228 | Ken Kemphues  [Rodriguez et al., 2017](#_ENREF_12) |
| *mex-5(ax3050[mcherry::mex-5])IV* | JH3296 | [Smith et al., 2016](#_ENREF_14) |
| *unc-119(ed3)III; tjls173[Ppie-1::gfp::air-1^R +^ unc-119(+)]* | SA378 | [Toya, Terasawa, Nagata, Iida, & Sugimoto, 2011](#_ENREF_16) |
| *unc-119(ed3)III; tjls188[Ppie-1 promoter-gfp::air-1^R^T201A + unc-119(+)]* | SA400 | [Toya, Terasawa, Nagata, Iida, & Sugimoto, 2011](#_ENREF_4) |
| *unc-119(ed3)III; axIs1933[pFM034, gfp::par-2 ^RNAi-resistant +^ unc-119(+)]* | JH2814 | [Motegi et al., 2011](#_ENREF_7) |
| *unc-119(ed3)III; axIs1936[pFM037, gfp::par-2 ^RNAi-resistant^(R163A) + unc-119(+)]* | JH2817 | [Motegi et al., 2011](#_ENREF_1) |
| *unc-119(ed3)III; axIs1934[pFM035, gfp::par-2 ^RNAi-resistant^(R183-5A) + unc-119(+)]* | JH2815 | [Motegi et al., 2011](#_ENREF_1) |
| *nmy-2(ne3409)I* | WM179 | [Pang et al., 2004](#_ENREF_11) |
| *juIs352[Pcol-19::gfp::moesin]* | [CZ14748](https://cgc.umn.edu/strain/CZ14748) | [Xu & Chisholm, 2011](#_ENREF_19) |
| *spd-2(or293)I* | EU780 | [O'Rourke et al., 2011](#_ENREF_10) |
| *unc-119(ed3)III; zuIs45[Pnmy-2::nmy-2::gfp + unc-119(+)]V* | [CZ14748](https://cgc.umn.edu/strain/CZ14748) | [Munro, Nance, & Priess, 2004](#_ENREF_8) |
| *spd-5(or213)I* | [EU](https://cgc.umn.edu/strain/CZ14748)856 | [Hamill, Severson, Carter, & Bowerman, 2002](#_ENREF_6) |
| *unc-119(ed3)III; itIs38[pAA1, Ppie-1::gfp::ph(PLC1delta1 PH) + unc-119(+)]* | [OD58](https://cgc.umn.edu/strain/CZ14748) | [Audhya et al., 2005](#_ENREF_1) |
| *fem-1(hc17)IV* | BA17 | [Wilson et al., 2006](#_ENREF_18) |
| *xsSi3[gfp::utrophin + Cbr-unc-119(+)]II; unc-119(ed3)III* | MG589 | [Tse et al., 2012](#_ENREF_17) |
| *such-1(t1668) unc-32(e189)/qC1 [dpy-19(e1259) glp-1(q339)]III; him-3(e1147)* | GZ759 | [Bezler & Gönczy, 2010](#_ENREF_2) |
| **genotype of original strains** | **strain** | **Reference** |
| *mcm-4(e1466) dpy-5(e61) I/hT2 [bli-4(e937) let-?(q782) qIs48] (I;III)* | JK2739 | Judith Kimble  https://cgc.umn.edu/strain/JK2739 |
| *zif-1(gk117)III;air-1(wow14[air-1::zf::gfp])V;wowEx34* | JLF189 | [Sallee, Zonka, Skokan, Raftrey, & Feldman, 2018](#_ENREF_13) |
| *mat-1(ax161)I; him-8(e1489) IV* | DS77 | [Golden et al., 2000](#_ENREF_5) |
| *unc-119(ed3) III; par-2(ok1723); ddIs239[pie-1::GFP::par-2(CAI 0.6) + unc-119(+)]* | TH415 | [Goehring, Hoege, Grill, & Hyman, 2011](#_ENREF_4) |
| **genotype of new strains generated in this study** | **strain** | **Reference** |
| *sas-7(is1[rfp::sas-7+loxP])III; glo-1(zu931)X* | GZ1522 | this study |
| *unc-119(ed3)III; isIs44[Ppie-1::gbp::mCherry::air-1 + unc-119(+)]* | GZ1232 | this study |
| *spd-2(is2(rfp::spd-2+loxP))I* | GZ1497 | this study |
| **genotype of crossed strains** | **strain** | **Reference** |
| *nmy-2(ges6(nmy-2::RFP-T+unc-119(+)))I; par-2(it328[gfp::par-2]) sas-7(or1940[gfp::sas-7])III* | GZ1476 | this study |
| *par-6(it319[par-6::gfp])I; par-2(it315[mcherry::par-2]) sas-7(is1[rfp::sas-7+loxP])III* | GZ1591 | this study |
| *par-2(it315[mcherry::par-2])III; tjls173[Ppie-1::gfp::air-1R + unc-119(+)]* | GZ1465 | this study |
| *par-2(it315[mcherry::par-2])III; tjls188[Ppie-1 promoter-gfp::air-1RT201A + unc-119(+)]* | GZ1466 | this study |
| *nmy-2(ges6(nmy-2::RFP-T+unc-119(+)))I; axIs1933[pFM034, gfp::par-2 RNAi-resistant + unc-119(+)]* | GZ1642 | this study |
| *nmy-2(ges6(nmy-2::RFP-T+unc-119(+)))I; axIs1936[pFM037, gfp::par-2 RNAi-resistant(R163A) + unc-119(+)]* | GZ1643 | this study |
| *spd-2(or293)I; par-2(it328[gfp::par-2]) sas-7(or1940[gfp::sas-7])III; zuIs45[Pnmy-2::nmy-2::gfp + unc-119(+)]V* | GZ1589 | this study |
| *par-2(it315[mcherry::par-2]) sas-7(is1[rfp::sas-7+loxP])III; zuIs45[Pnmy-2::nmy-2::gfp + unc-119(+)]V* | GZ1588 | this study |
| *spd-5(or213)I, par-2(it315[mcherry::par-2]) sas-7(is1[rfp::sas-7+loxP])III; zuIs45[Pnmy-2::nmy-2::gfp + unc-119(+)]V* | GZ1617 | this study |
| *isIs44[Ppie-1::gbp::mCherry::air-1 + unc-119(+)] itIs38[pAA1, Ppie-1::gfp::ph(PLC1delta1 PH) + unc-119(+)]* | GZ1233 | this study |
| *par-2(it328[gfp::par-2])III; such-1(t1668)/mcm-4(e1466) dpy-5(e61) I/hT2 [bli-4(e937) let-?(q782) qIs48] (I;III)* | GZ1515 | this study |
| *nmy-2(ges6(nmy-2::RFP-T+unc-119(+)))I*; par-2(it328[gfp::par-2]) sas-7(or1940[gfp::sas-7])III; *fem-1(hc17)IV* | GZ1546 | this study |
| *nmy-2(ne3409)I; xsSi3[gfp::utrophin + Cbr-unc-119(+)]II* | GZ1619 | this study |
| *nmy-2(ne3409)I; par-2(it328[gfp::par-2]) sas-7(or1940[gfp::sas-7])III* | GZ1618 | this study |
| *par-2(it315[mcherry::par-2]); isIs44[Ppie-1::gbp::mCherry::air-1 + unc-119(+)]* | GZ1622 | this study |
| *mat-1(ax161)I; par-2(it315(mCherry::par-2))III; sas-7(is1[rfp::sas-7+loxP])III; ltIs25 [pAZ132: unc-119 (+) pie-1-gfp- tba-2]* | GZ1615 | this study |
| *unc-119(ed3)III; nBsIs20*[pNP99:unc-119(+)tbb-1p-mCherry-tbb-2-tbb-2 UTR*]; unc-119(ed3)III; tjls173[Ppie-1::gfp::air-1^R +^unc-119(+)]* | GZ1226 | this study |
| *unc-119(ed3)III; nBsIs20*[pNP99:unc-119(+)tbb-1p-mCherry-tbb-2-tbb-2 UTR*]; unc-119(ed3)III; tjls188[Ppie-1 promoter-gfp::air-1^R^T201A + unc-119(+)]* | GZ1648 | this study |
| *zif-1(gk117)III;air-1(wow14[air-1::zf::gfp])V;wowEx34; unc-119(ed3)III; nBsIs20*[pNP99:unc-119(+)tbb-1p-mCherry-tbb-2-tbb-2 UTR*]* | GZ1698 | this study |

**References**

Audhya, A., Hyndman, F., McLeod, I. X., Maddox, A. S., Yates, J. R., 3rd, Desai, A., & Oegema, K. (2005). A complex containing the Sm protein CAR-1 and the RNA helicase CGH-1 is required for embryonic cytokinesis in Caenorhabditis elegans. *J Cell Biol, 171*(2), 267-279. doi: 10.1083/jcb.200506124

Bezler, A., & Gönczy, P. (2010). Mutual antagonism between the anaphase promoting complex and the spindle assembly checkpoint contributes to mitotic timing in Caenorhabditis elegans. *Genetics, 186*(4), 1271-1283. doi: 10.1534/genetics.110.123133

Brenner, S. (1974). The genetics of Caenorhabditis elegans. *Genetics, 77*(1), 71-94.

Goehring, N. W., Hoege, C., Grill, S. W., & Hyman, A. A. (2011). PAR proteins diffuse freely across the anterior-posterior boundary in polarized C. elegans embryos. *J Cell Biol, 193*(3), 583-594. doi: 10.1083/jcb.201011094

Golden, A., Sadler, P. L., Wallenfang, M. R., Schumacher, J. M., Hamill, D. R., Bates, G., Shakes, D. C. (2000). Metaphase to anaphase (mat) transition-defective mutants in Caenorhabditis elegans. *J Cell Biol, 151*(7), 1469-1482.

Hamill, D. R., Severson, A. F., Carter, J. C., & Bowerman, B. (2002). Centrosome maturation and mitotic spindle assembly in C. elegans require SPD-5, a protein with multiple coiled-coil domains. *Dev Cell, 3*(5), 673-684.

Motegi, F., Zonies, S., Hao, Y., Cuenca, A. A., Griffin, E., & Seydoux, G. (2011). Microtubules induce self-organization of polarized PAR domains in Caenorhabditis elegans zygotes. *Nat Cell Biol, 13*(11), 1361-1367. doi: 10.1038/ncb2354

Munro, E., Nance, J., & Priess, J. R. (2004). Cortical flows powered by asymmetrical contraction transport PAR proteins to establish and maintain anterior-posterior polarity in the early C. elegans embryo. *Dev Cell, 7*(3), 413-424. doi: 10.1016/j.devcel.2004.08.001

Nishikawa, M., Naganathan, S. R., Julicher, F., & Grill, S. W. (2017). Controlling contractile instabilities in the actomyosin cortex. *Elife, 6*. doi: 10.7554/eLife.19595

O'Rourke, S. M., Carter, C., Carter, L., Christensen, S. N., Jones, M. P., Nash, B., Bowerman, B. (2011). A survey of new temperature-sensitive, embryonic-lethal mutations in C. elegans: 24 alleles of thirteen genes. *PLoS One, 6*(3), e16644. doi: 10.1371/journal.pone.0016644

Pang, K. M., Ishidate, T., Nakamura, K., Shirayama, M., Trzepacz, C., Schubert, C. M., Mello, C. C. (2004). The minibrain kinase homolog, mbk-2, is required for spindle positioning and asymmetric cell division in early C. elegans embryos. *Dev Biol, 265*(1), 127-139.

Rodriguez, J., Peglion, F., Martin, J., Hubatsch, L., Reich, J., Hirani, N., Goehring, N. W. (2017). aPKC Cycles between Functionally Distinct PAR Protein Assemblies to Drive Cell Polarity. *Dev Cell, 42*(4), 400-415 e409. doi: 10.1016/j.devcel.2017.07.007

Sallee, M. D., Zonka, J. C., Skokan, T. D., Raftrey, B. C., & Feldman, J. L. (2018). Tissue-specific degradation of essential centrosome components reveals distinct microtubule populations at microtubule organizing centers. *PLoS Biol, 16*(8), e2005189. doi: 10.1371/journal.pbio.2005189

Smith, J., Calidas, D., Schmidt, H., Lu, T., Rasoloson, D., & Seydoux, G. (2016). Spatial patterning of P granules by RNA-induced phase separation of the intrinsically-disordered protein MEG-3. *Elife, 5*. doi: 10.7554/eLife.21337

Sugioka, K., Hamill, D. R., Lowry, J. B., McNeely, M. E., Enrick, M., Richter, A. C., Bowerman, B. (2017). Centriolar SAS-7 acts upstream of SPD-2 to regulate centriole assembly and pericentriolar material formation. *Elife, 6*. doi: 10.7554/eLife.20353

Toya, M., Terasawa, M., Nagata, K., Iida, Y., & Sugimoto, A. (2011). A kinase-independent role for Aurora A in the assembly of mitotic spindle microtubules in Caenorhabditis elegans embryos. *Nat Cell Biol, 13*(6), 708-714. doi: 10.1038/ncb2242

Tse, Y. C., Werner, M., Longhini, K. M., Labbe, J. C., Goldstein, B., & Glotzer, M. (2012). RhoA activation during polarization and cytokinesis of the early Caenorhabditis elegans embryo is differentially dependent on NOP-1 and CYK-4. *Mol Biol Cell, 23*(20), 4020-4031. doi: 10.1091/mbc.E12-04-0268

Wilson, M. A., Shukitt-Hale, B., Kalt, W., Ingram, D. K., Joseph, J. A., & Wolkow, C. A. (2006). Blueberry polyphenols increase lifespan and thermotolerance in Caenorhabditis elegans. *Aging Cell, 5*(1), 59-68. doi: 10.1111/j.1474-9726.2006.00192.x

Xu, S., & Chisholm, A. D. (2011). A Galphaq-Ca(2)(+) signaling pathway promotes actin-mediated epidermal wound closure in C. elegans. *Curr Biol, 21*(23), 1960-1967. doi: 10.1016/j.cub.2011.10.050
