# Supplementary material for: Aurora A depletion reveals centrosome-independent polarization mechanism in Caenorhabditis elegans
Source: eLife. 2019 Feb 26;8:e44552. doi: 10.7554/eLife.44552 (PMC6417861; doi:10.7554/eLife.44552)
Supplement: Supplementary file 2. [file elife-44552-supp2.docx]

| **Table S2: Statistical Analysis** | | | | | | |
| --- | --- | --- | --- | --- | --- | --- |
| **p-values** | *** < 0.001 | ** < 0.01 | * <0.05 | ns >0.05 |  |  |
|  |  |  |  |  |  |  |
| **Figure** | **worm strain** | **comparison** | **posterior vs non-posterior** | **anterior vs non-anterior** | **bipolar vs non-bipolar** | **no polarity vs polarized** |
| **Fig.1 C** | RFP::NMY-2; GFP::PAR-2; GFP::SAS-7 | control vs *air-1(RNAi)* | <0.0001 | 0.0263 | <0.0001 | 0.5485 |
| **Fig. 1 D** | N2 | control vs *air-1(RNAi)* | <0.0001 | 0.0071 | <0.0001 | >0.999 |
| **Fig. 1 H** | GFP::PAR-6, mCherry::PAR-2; RFP::SAS-7 | control vs *air-1(RNAi)* | >0.999 | <0.0001 | 0.0003 | >0.999 |
| **Fig. 1 J** | GFP::PKC-3 | control vs *air-1(RNAi)* | 0.2874 | <0.0001 | <0.0001 | >0.999 |
| **Fig. 1 L** | mCherry::MEX-5 | control vs *air-1(RNAi)* | 0.2663 | <0.0001 | 0.0006 | >0.999 |
| **Fig. 1N** | GFP::AIR-1 WT R | control vs *air-1(RNAi)* | 0.0006 | 0.1268 | 0.0079 | >0.999 |
| **Fig. 1N** | GFP::AIR-1 T201A R | control vs *air-1(RNAi)* | <0.0001 | >0.999 | <0.0001 | >0.999 |
| **Fig. 2F** | RFP::NMY-2; GFP::PAR-2; GFP::SAS-7 | *rga-3(RNAi)* vs *air-1(RNAi)+rga-3(RNAi)* | <0.0001 | 0.0253 | <0.0001 | >0.999 |
| **Fig. 2H** | *nmy-2(ne3409*); GFP::PAR-2; GFP::SAS-7 | control vs *air-1(RNAi)* | <0.0001 | >0.999 | <0.0001 | >0.999 |
| **Fig. 2 J** | GFP::PAR-2 WT R | control vs *air-1(RNAi)* | <0.0001 | 0.0171 | 0.0001 | >0.999 |
| **Fig. 2 K** | GFP::PAR-2 R163A R + *par-2(RNAi)* | control vs *air-1(RNAi)* | <0.0001 | >0.999 | >0.999 | <0.0001 |
| **Fig. 6C** | GBP::mCherry::AIR-1; mCherry::PAR-2 | *air-1(RNAi)+spd-2(RNAi) vs air-1(RNAi)+spd-2(RNAi) +*GFP::PH | 0.0174 | 0.144 | 0.2685 | <0.0001 |
| **Fig. 6E** | *mat-1(ax161) + air-1(RNAi)* | control vs *air-1(RNAi)* 2h | >0.999 | 0.0061 | 0.0061 | >0.999 |
| **Fig. 6F** | *mat-1(ax161) + air-1(RNAi)* | control vs *air-1(RNAi)* 12h | >0.999 | >0.999 | >0.999 | >0.999 |
| **Fig. 7B** | GFP::NMY-2; mCherry::PAR-2; RFP::SAS-7 | control vs *spd-2(or293)* | <0.0001 | 0.5079 | <0.0001 | >0.999 |
| **Fig. 7B** | GFP::NMY-2; mCherry::PAR-2; RFP::SAS-8 | control vs *spd-5(or213)* | <0.0001 | 0.501 | <0.0001 | >0.999 |
| **Fig. 7B** | GFP::NMY-2; mCherry::PAR-2; RFP::SAS-9 | control vs *tbg-1(RNAi)* | >0.999 | >0.999 | >0.999 | >0.999 |
| **Fig. 7E** | RFP::NMY-2; GFP::PAR-2; GFP::SAS-7; *fem-1(hc17)* | centrosomes vs no centrosomes | <0.0001 | 0.0055 | 0.0069 | >0.999 |
| **Fig. 7J** | RFP::NMY-2; GFP::PAR-2; GFP::SAS-7 +*air-1(RNAi)* | posterior vs anterior sperm entry | >0.999 | 0.6823 | 0.7166 | >0.999 |
| **Fig. 7L** | GBP::mCherry::AIR-1; mCherry::PAR-2 | control vs *air-1(RNAi)* | 0.0208 | >0.999 | 0.0208 | >0.999 |
| **Fig. 7L** | GBP::mCherry::AIR-1; mCherry::PAR-2 | *air-1(RNAi)* vs *air-1(RNAi)+spd-2(RNAi)* | 0.0036 | 0.0957 | 0.2377 | >0.999 |
| **Fig. 7L** | GBP::mCherry::AIR-1; mCherry::PAR-2 | *air-1(RNAi)+spd-2(RNAi) vs air-1(RNAi)+spd-2(RNAi)* +male GFP::SAS-7 | 0.0174 | 0.144 | 0.2685 | >0.999 |
| **Fig. 7L** | GBP::mCherry::AIR-1; mCherry::PAR-2 | *air-1(RNAi) vs air-1(RNAi)+spd-2(RNAi)* +male GFP::SAS-7 | >0.999 | >0.999 | >0.999 | >0.999 |
|  |  |  |  |  |  |  |
|  |  |  |  |  |  |  |
| **Fig. 3B** | RFP::NMY-2; GFP::PAR-2; GFP::SAS-7 number PAR-2 domains | control vs *air-1(RNAi)* | <0.0001 |  |  |  |
| **Fig. 3C** | RFP::NMY-2; GFP::PAR-2; GFP::SAS-7 PAR-2 domain localization | control vs *air-1(RNAi)* | 0.0059 |  |  |  |
